# Stability, Bifurcation and Chaos Analysis of Vector-Borne Disease Model with Application to Rift Valley Fever

**DOI:** 10.1371/journal.pone.0108172

**Published:** 2014-10-01

**Authors:** Sansao A. Pedro, Shirley Abelman, Frank T. Ndjomatchoua, Rosemary Sang, Henri E. Z. Tonnang

**Affiliations:** 1 School of Computational and Applied Mathematics, University of the Witwatersrand, Johannesburg, South Africa; 2 Modelling, International Center of Insect Physiology and Ecology, Nairobi, Kenya; 3 Human Health, International Center of Insect Physiology and Ecology, Nairobi, Kenya; 4 Departmento de Matemática e Informática, Universidade Eduardo Mondlane, Maputo, Mozambique; 5 Departement de Physique, Universite de Yaoundé I, Yaoundé, Cameroun; Institut Pasteur, France

## Abstract

This paper investigates a RVF epidemic model by qualitative analysis and numerical simulations. Qualitative analysis have been used to explore the stability dynamics of the equilibrium points while visualization techniques such as bifurcation diagrams, Poincaré maps, maxima return maps and largest Lyapunov exponents are numerically computed to confirm further complexity of these dynamics induced by the seasonal forcing on the mosquitoes oviposition rates. The obtained results show that ordinary differential equation models with external forcing can have rich dynamic behaviour, ranging from bifurcation to strange attractors which may explain the observed fluctuations found in RVF empiric outbreak data, as well as the non deterministic nature of RVF inter-epidemic activities. Furthermore, the coexistence of the endemic equilibrium is subjected to existence of certain number of infected *Aedes* mosquitoes, suggesting that *Aedes* have potential to initiate RVF epidemics through transovarial transmission and to sustain low levels of the disease during post epidemic periods. Therefore we argue that locations that may serve as RVF virus reservoirs should be eliminated or kept under control to prevent multi-periodic outbreaks and consequent chains of infections. The epidemiological significance of this study is: (1) low levels of birth rate (in both *Aedes* and *Culex*) can trigger unpredictable outbreaks; (2) *Aedes* mosquitoes are more likely capable of inducing unpredictable behaviour compared to the *Culex*; (3) higher oviposition rates on mosquitoes do not in general imply manifestation of irregular behaviour on the dynamics of the disease. Finally, our model with external seasonal forcing on vector oviposition rates is able to mimic the linear increase in livestock seroprevalence during inter-epidemic period showing a constant exposure and presence of active transmission foci. This suggests that RVF outbreaks partly build upon RVF inter-epidemic activities. Therefore, active RVF surveillance in livestock is recommended.

## Introduction

Rift Valley fever (RVF) virus, a member of the genus phlebovirus and family Bunyaviridae, which has been isolated from at least 40 mosquito species in the field [1], infects both wild and domestic animals and humans. The RVF epizootics and epidemics are closely linked to the occurrence of the warm phase of the El Nino/Southern Oscillation (ENSO) phenomenon [2]. This phenomenon is characterized by elevated Indian Ocean temperatures which lead to heavy rainfall and flooding of habitats suitable for the production of immature *Aedes* and *Culex* mosquitoes that serve as the primary RVF virus (RVFV) vectors in East Africa [3], [4]. Studies have shown that the life cycle of RVFV has distinct endemic and epidemic cycles. During the endemic cycle the virus persists during dry season/inter-epizootic periods through vertical transmission in *Aedes* mosquito eggs [3]. *Aedes* eggs need to be dry for several days before they can mature. After maturing, they hatch during the next flooding event large enough to cover them with water [5],[6]. The eggs have high desiccation resistance and can survive dry conditions in a dormant form for months to years. At the beginning of the rainy season, *Aedes* mosquitoes quickly multiply into large numbers before declining due to the need for dry conditions for egg maturation [9]. There can be a second peak in mosquito densities at the end of the rainy season if there is a gap in rainfall for several days [5]. When these mosquitoes lay their eggs in flooded areas (including dambos), transovarially infected adults may emerge and transmit RVFV to nearby domestic animals, including sheep, goats, cattle, and camels. High viremias in these animals may then lead to the infection of secondary arthropod vector species including various *Culex* species [7].

Epizootic/epidemic cycles are driven by the subsequent elevation of various *Culex* mosquito populations, which serve as excellent secondary vectors if immature mosquito habitats remain flooded for a long enough period [4]. Their eggs require water to mature and hatch and the mosquitoes survive the dry season in adult form and during the rainy season, the population of *Culex* mosquitoes reaches a maximum towards the end of the season [9]. The propagation of these secondary vectors may spread the virus to additional infection in animal and human, causing an outbreak. The disease is known to occur in outbreaks that come in cycles of 5–15 years in the Eastern Africa region and the Horn of Africa [10].

We observe that RVF outbreaks are highly linked to seasonal variations on rainfall, which is in turn reflected through seasonal fluctuations in mosquito population densities. *Aedes* eggs require water to hatch and dry condition for maturation, and at the beginning of the rainy season quickly grow to large numbers while *Culex* eggs require water to mature and hatch, and survive dry season in adult form and during the rainy season reach maximum numbers towards the end of the season. Thus, fluctuations in both seasons (wet and dry) favour the complex dynamics of both mosquito species. Hence the complexity observed on the dynamics of RVF virus transmission and maintenance.

The interplay between the internal nonlinear dynamic of ecological systems and various external factors that affect them, makes understanding of population fluctuation a unique problem [11].

Mathematical models have been developed in order to provide a better understanding of the nature and dynamics of the transmission and persistence of the disease, as well as predict outbreaks and simulate the impact of control strategies [9], . Most of these models considered constant mosquito oviposition rates, ignoring effects of seasonal fluctuations in the mosquito population size. Furthermore, some have ignored the effects of vertical transmission and secondary vectors [18] and some only considered *Aedes* species [9]. Temperature, rainfall and humidity have great influence in all stages of mosquito development from the emergence and viability of eggs, to the size and longevity of adults [19], [20]. Recently, Mpeshe et al. [21] modified their previous study [18] to include vertical transmission in *Aedes* species and climate-driven parameters. These models provide important insights but do not investigate the stability dynamics and attractors structures of the model when there are external forces in the density of vector populations.

The most common manifestation of external forcing is through seasonality including both natural (e.g. the occurrence of the warm phase of the El Nino/Southern oscillation phenomenon) and induced (e.g human deforestation or human pollution).

Studies for understanding dynamical consequences of regular and stochastic external forcing are still ongoing but poorly understood [22]–[25]. To the best of our knowledge, no systematic investigation of stability and attractor structures of a realistic RVF model comprising two populations of mosquitoes (*Aedes* and *Culex*) and one livestock host population with two infected classes (asymptomatic and symptomatic) and seasonal variation on mosquito oviposition rates has been carried out.

Based on the model proposed by Gaff et al. [12], we investigate a two vector and one host epidemic model, to capture the dynamical behaviour of both the disease free and endemic equilibria, the effects of seasonality on mosquito oviposition rates 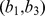, parametrized by 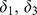 and effects of asymptomatic class in livestock (parametrized by 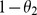). We prove existence and global stability of both the disease-free and the endemic equilibria in the absence of secondary vectors 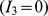, as well as the existence and local stability of both disease free and endemic equilibrium points of the overall model. We then investigate the structures of model attractors through bifurcation analysis, taking as bifurcation parameters 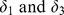 the strengths of seasonality of mosquito oviposition rates. The bifurcation diagrams with simultaneous variation of seasonal forcing on the oviposition rates of the two mosquito species reveal the complexity induced by their interactions. The understanding of possible state space scenarios through bifurcation analysis is helpful for understanding RVF epidemiological data with its seasonality aspects. To obtain robust analysis we then compute the largest Lyapunov exponents, Poincaré maps and maxima return maps.

The section methods gives a detailed description of the model and its parameters. In section results the model is used to study the dynamic behaviour of the disease stability and bifurcation analysis. Simulations are performed to investigate model dependence on initial condition and attractors structures of the model applying an external forcing on mosquito's oviposition rates.

## Methods

Gaff et al. [12] proposed a one host and two vectors population model for RVF with vertical transmission in *Aedes* vectors to study the transmission of RVF and the impact of vertical transmission on the persistence of the disease. Chitnis et al. [9] analysed a RVF model with vertical transmission for *Aedes* mosquitoes and included asymptomatic class for livestock and removed one population of mosquitoes.

The model presented in this paper adopts a similar structure as in Gaff et al. [12]. We introduce an asymptomatic class for livestock [9], because for many species of livestock, RVF virus infection are frequently subclinical [26], [27]. As the main purpose of this study is to study the dynamic behaviour of the disease, influenced by changes in climate and oscillation of rainfall, we include seasonal variation in the oviposition rates of both *Aedes* and *Culex* mosquitoes.

We divide the livestock population into four classes: susceptible, 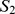, asymptomatic, 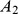, infectious, 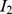, and recovered (immune), 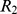. Livestock enter the susceptible class through birth (at a constant rate). Birth rates are important because after an outbreak, herd immunity can reach 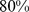 and the proportion of susceptible livestock must be renewed through birth or movement before another outbreak can occur [28]. When an infectious mosquito bites a susceptible animal, there is a finite probability that the animal becomes infected. Since the duration of the latent period in cattle is small relative to their life span, we do not model the exposed stage. Many adult cattle do not exhibit clinical signs apart from abortion of foetuses [6], [26], thus, include an asymptomatic class for infectious animals that transmit the virus at a lower rate than those with acute clinical symptoms. After being successfully infected by an infectious *Aedes* and/or *Culex* mosquito, livestock move from the susceptible class 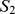 to either the infected symptomatic 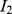 or asymptomatic 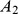 class. After some time, the symptomatic and asymptomatic livestock recover and move to the recovered class, 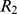. The recovered livestock have immunity to the disease for life. Cattle leave the population through a per capita natural death rate and through a per capita disease-induced death rate only for symptomatic livestock. The size of the livestock population is given by 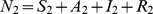.

We divide the *Aedes* and *Culex* mosquitoes population into three classes: susceptible, 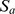, exposed, 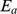, and infectious, 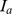. The subscripts 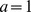 and 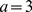 represent *Aedes* and *Culex* mosquitoes, respectively. Female mosquitoes (we do not include male mosquitoes in our model because only female mosquitoes bite animals for blood meals) enter the susceptible class through birth. The virus enters a susceptible mosquito, 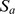, with finite probability, when the mosquito bites an infectious animal and the mosquito moves to the exposed class, 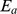. After some period of time, depending on the ambient temperature and humidity [29], the mosquito moves from the exposed class to the infectious class, 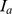. To reflect the vertical transmission in the *Aedes* species, compartments for uninfected 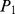 and infected 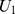 eggs are included. As the *Culex* species cannot transmit RVF vertically, only uninfected eggs 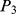 are included. Mosquitoes once infected remain infectious during their lifespan. Mosquitoes leave the population through a per capita natural death rate. The size of each adult mosquito population is 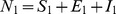 for adult *Aedes* mosquitoes and 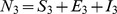 for adult *Culex* mosquitoes. The three populations are modelled with carrying capacity 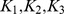, for *Aedes*, livestock and *Culex* respectively. While in [12], the total number of mosquito bites on cattle depends on the number of mosquitoes, in our model, the total number of bites varies with both the cattle and mosquito population sizes. This allows a more realistic modelling of situations where there is a high ratio of mosquitoes to cattle, and where cattle availability to mosquitoes is reduced through control interventions [9].

### 0.1 Mathematical Model

**Figure 1:**
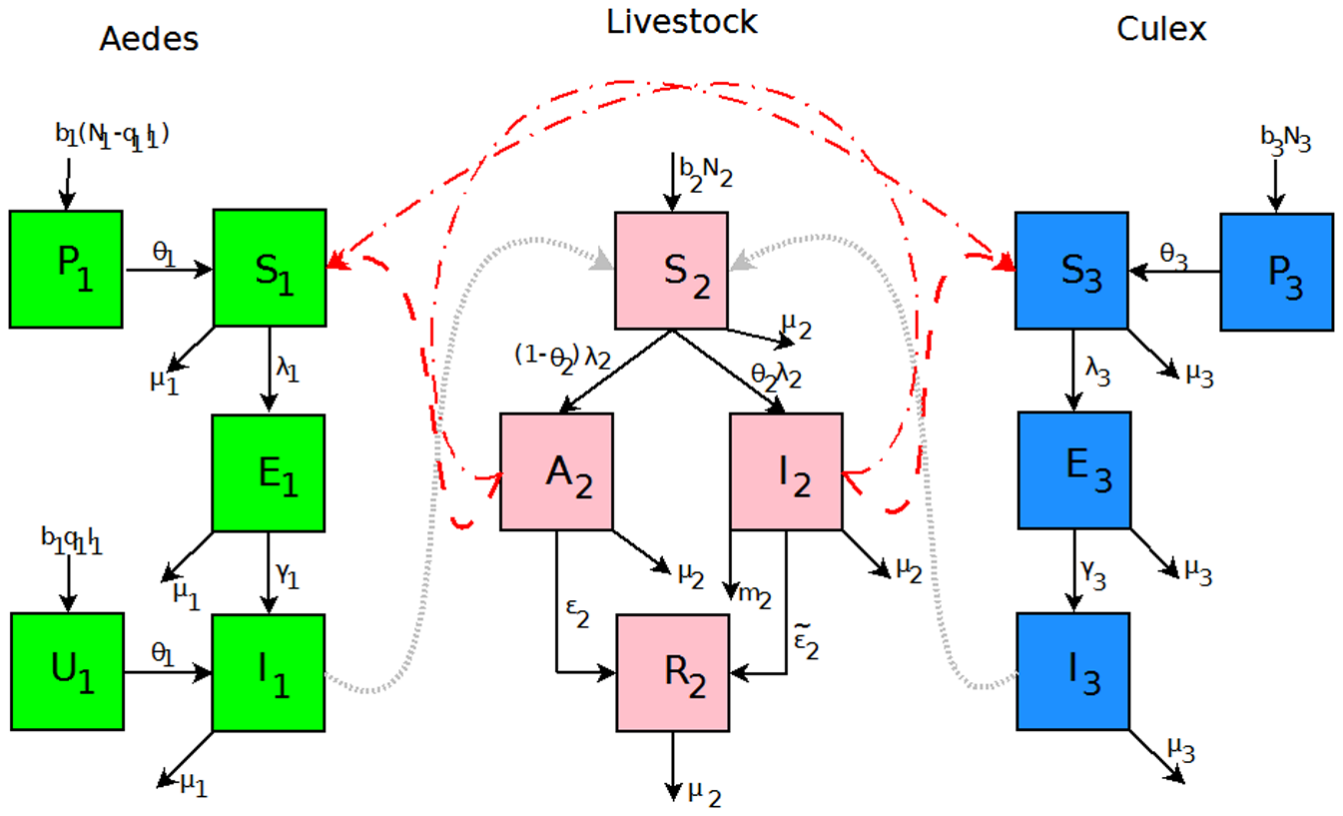
Flow diagram of RVFV transmission with each species, namely, *Aedes* mosquitoes, *Culex* mosquitoes and livestock (the solid lines represent the transition between compartments and the dash lines represent the transmission between different species).

The state variables in Table 1 and parameters in Table 2 for the RVF model (Figure 1) satisfy the following system of equations:

**Table 1:** State variables for the model system (1,2,3).

Variable	Description
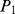	Number of uninfected *Aedes* mosquito eggs
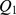	Number of infected *Aedes* mosquito eggs
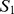	Number of susceptible *Aedes* mosquitoes
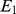	Number of exposed *Aedes* mosquitoes
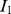	Number of infected *Aedes* mosquitoes
	
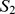	Number of susceptible livestock
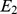	Number of exposed livestock
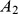	Number of asymptomatic livestock
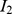	Number of infected livestock
	
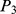	Number of uninfected *Culex* mosquito eggs
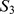	Number of susceptible *Culex* mosquitoes
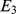	Number of exposed *Culex* mosquitoes
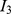	Number of infected *Culex* mosquitoes

**Table 2:** Parameters description for the RVF model (1,2,3).

Parameter	Values	References	Parameters description and their dimensions
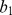	0.06	[9], [13]	Per capita birth/death rate of *Aedes* mosquito species, Day^−1^
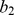	0.0022	[12]	Per capita birth/death rate of livestock, Day^−1^
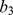	0.06	[9], [13]	Per capita birth/death rate of *Culex* mosquito species, Day^−1^
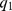	0.1	[14]	Probability of vertical transmission from an infectious *Aedes* mosquito mother
			to its eggs, dimensionless
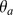	0.20	Assumed	Development rate of mosquitoes, Day^−1^, where 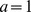 and 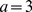
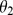	0.6	[6], [9]	Probability of an infected host moving to the symptomatic stage, dimensionless
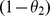	0.4	[6], [9]	Probability of an infected host moving to the asymptomatic stage, dimensionless
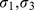	0.33	[5], [9]	Number of times one *Aedes*, *Culex* mosquito would want to bite a host per Day, if it were freely available. This is a function of the mosquito's gonotrophic cycle (the amount of time a mosquito requires to produce eggs) and its preference for livestock blood, Day^−1^
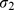	19	[9]	The maximum number of mosquito bites a host can sustain per Day. This is a function of the host's exposed surface area, the efforts it takes to prevent mosquito bites (such as switching its tail), and any vector control interventions in place to kill mosquitoes encountering hosts or preventing bites, Day^−1^
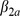	0.21	[6], [9]	Probability of transmission of infection from an infectious mosquito to a susceptible host given that a contact between the two occurs, dimensionless, where 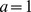 and 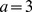
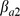	0.7,0.15	[6], [9]	Probability of transmission of infection from an infectious host to a susceptible mosquito given that a contact between the two occurs, dimensionless, where 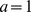 and 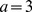
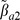	0.30	[6], [9]	Probability of transmission of infection from an asymptomatic host to a susceptible mosquito given that a contact between the two occurs, dimensionless
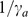	6	[12], [15]	is the average duration of the mosquitoes latent period, Days, where 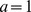 and 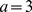
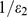	4	[5], [9], [16]	is the average duration of the infectious period 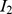 , Days
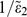	4	[9], [13], [16]	is the average duration of the infectious asymptomatic period, Day^−1^
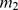	0.1	[9], [13], [16]	Per capita disease-induced death rate for livestock, Day^−1^
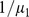	20	[9], [13]	Lifespan of *Aedes* mosquitoes, Days
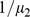	2190	[12]	Lifespan of livestock animals, Days
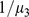	20	[9], [13]	Lifespan of *Culex* mosquitoes, Days

**Aedes**

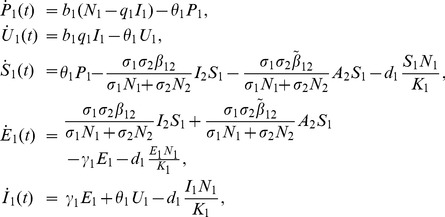
(1)


**Livestock**

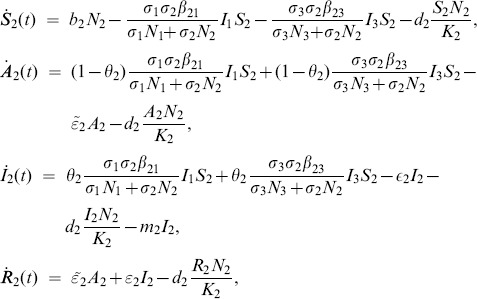
(2)


**Culex**

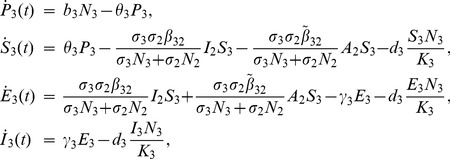
(3)where from the model flowchart in Fig.1, 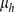 for 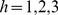 represents the natural death rate given by 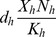, 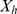 representing each compartment of every species in the model, with
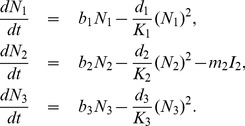
(4)


Following the approach in [9], 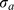, where 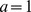 for *Aedes* and 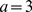 for *Culex* is the rate at which a mosquito would like to bite livestock (related to the gonotrophic cycle length), and 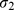 is the maximum number of bites that an animal can support per unit time (through physical availability and any intervention measures on livestock taken by humans). Then, 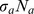 is the total number of bites that the mosquitoes would like to achieve per unit time and 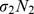 is the availability of livestock. Thus, the total number of mosquito-livestock contacts is half the harmonic mean of 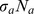 and 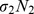,
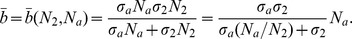



In addition to having the correct limits at zero and infinity, this form also meets the necessary criteria that 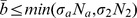 where 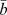 is the total number of bites per unit time. The total number of mosquito-livestock contacts depends on the populations of both species. We define 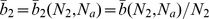 as the number of bites per livestock per unit time, and 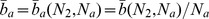 as the number of bites per mosquito per unit time.

We defined the force of infection from mosquitoes to livestock, 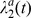, as the product of the number of mosquito bites that one animal has per unit time, 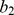, the probability of disease transmission from the mosquito to the animal, 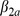, and the probability that the mosquito is infectious, 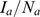. We define the force of infection from livestock to mosquitoes, 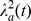, as the force of infection from infectious (symptomatic and asymptomatic) livestock. This is expressed as the number of livestock bites one mosquito has per unit time, 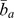; the probability of disease transmission from an infected (asymptomatic) animal to the mosquito, 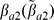; and the probability that the animal is infectious, 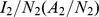. Therefore the forces of infection are given by:
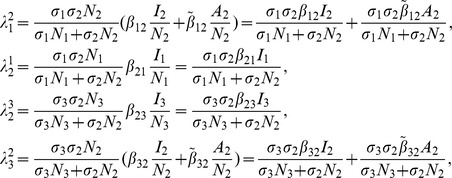



The model system (1,2,3) is biologically relevant (solutions are positive) in the set
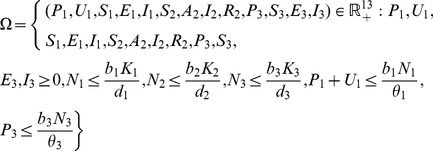
(5)


**Lemma 1.** *The model system (1,2,3) is well-posed in* 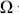 *which is invariant and attracting.*

**Proof 1.** *When* 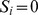 *for* 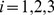 *then* 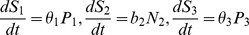 *that is* 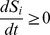 *for* 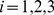 *for* 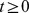.

*Similarly, when* 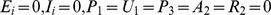 *for* 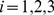 *we have* 
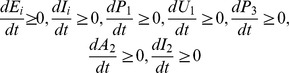
 *. If* 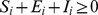 *for* 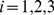 *and* 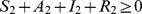 *we have* 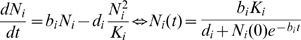 *for* 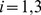 *and we show that for* 
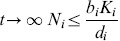
 *for* 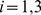 *.*

*Similarly, if* 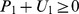 *we can show that* 
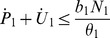
 *and* 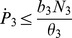 *for* 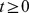 *. Thus, the solution remain in the feasible region* 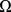 *if it starts in this region.*

## Results

### 0.2 Basic Reproduction Number

For epidemiology models, a quantity, 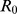 is derived to assess the stability of the disease free equilibrium [12]. 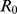 represents the the number of individuals infected by a single infected individual during his or her entire infectious period, in a population which is entirely susceptible [30]. When 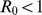, if a disease is introduced, there are insufficient new cases per case, and the disease cannot invade the population. When 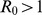, the disease may become endemic; the greater 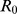 is above 1, the less likely stochastic fade out of the disease can occur. To compute this threshold we use the next generation operator approach, as described by Diekmann et al. [31] and van den Driessche and Watmough [32] as well as to describe the conditions for which the disease-free equilibrium points lose stability.

Since the model incorporates both vertical and horizontal transmission, 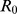 for the system is the sum of the 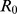 values for each mode of transmission determined separately [33],
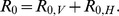



To compute each component of 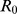, the model equations in vector form are the difference between the rate of new infection in compartment 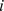, 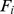 and the rate of transfer between compartment 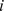 and all other compartments due to other processes, 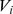 [32], (see Appendix S1). Then, 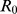 is given by
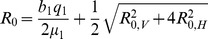
(6)where 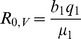 and
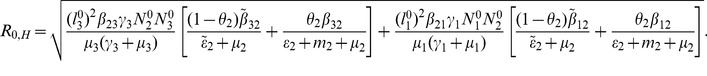
(7)


### 0.3 Basic Reproduction Number for periodic environment

In periodic environment, the basic reproduction number is the generalization of the 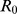 in non periodic environment. It is known as the transmissibility number 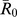, which is defined as the average number of secondary cases arising from the introduction of a single infectious individual into a completely susceptible population at a random time of the year [34]. Thus, 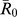 is defined through the spectral radius of a linear integral operator on a space of periodic functions, given by the integral operator 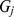 (see Appendix S1),
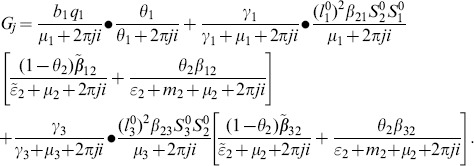
(8)


As proposed by Bacaer [36], the transmissibility number 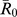 is given by
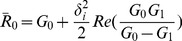
(9)where Re(.) is the real part of (.). 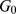 is the basic reproduction number for the non-seasonal model, obtained when 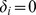.

The size of 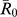 is reduced compared to 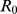 when oviposition rates are constant, and this makes it slightly difficult for the virus to invade the population with such fluctuations on the transmission rates [36].

From 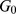 the following sub-reproduction numbers 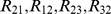 can be obtained: 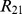 is the number of new infections in livestock from one infected *Aedes* mosquito and is given by
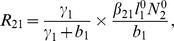
representing the product of the probability that the *Aedes* mosquito survives the exposed stage 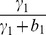, the number of bites on livestock per mosquito 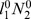, the probability of transmission per bite 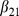, and the infectious lifespan of *Aedes* mosquito 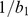.


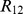
 is the number of new infections in *Aedes* mosquitoes from one infected (asymptomatic or symptomatic) animal, and is given by the weighted sum of new infections resulting from asymptomatic and symptomatic livestock
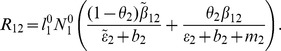



This is the product of the number of bites an animal receives 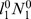, the probability of transmission per bite (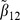 for an asymptomatic animal and 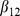 for symptomatic animal), and the duration of the infective period (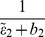 for an asymptomatic animal and 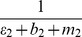 for symptomatic animal) weighted by the probability that an animal either becomes asymptomatic or symptomatic upon infection.


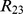
 is the number of new infections in livestock from one infected *Culex* mosquito and is given by
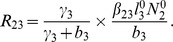



This is the product of the probability that the *Culex* mosquito survives the exposed stage 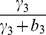, the number of bites on livestock per mosquito 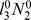, the probability of transmission per bite 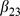, and the infectious lifespan of *Culex* mosquito 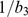.


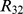
 is the number of new infections in *Culex* mosquitoes from an infected (asymptomatic or symptomatic) animal and is given by the weighted sum of new infections resulting from asymptomatic and symptomatic livestock
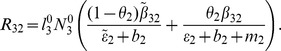



This is the product of the number of bites one animal receives 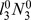, the probability of transmission per bite (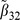 for an asymptomatic animal and 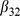 for symptomatic animal), and the duration of the infective period (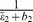 for an asymptomatic animal and 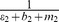 for symptomatic animal) weighted by the probability that an animal either becomes asymptomatic or symptomatic upon infection.

If 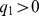, 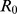 increases because vertical transmission directly increases the number of infectious mosquitoes and indirectly increases the transmission from livestock to mosquitoes and back to livestock.

### 0.4 Stability analysis

The computation of the equilibria for model system (1,2,3) yields, respectively: the disease-free equilibrium (DFE),
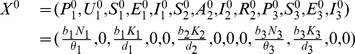
(10)and the endemic equilibrium (EE)
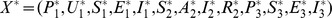
where
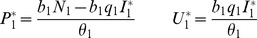
(11)




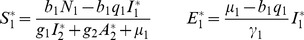
(12)

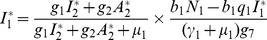
(13)

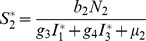
(14)

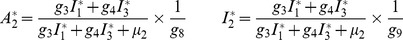
(15)

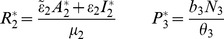
(16)

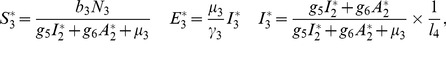
(17)



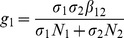
, 
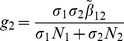
, 
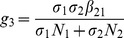
, 
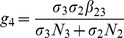
, 
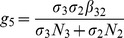
, 
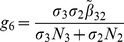
, 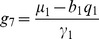, 
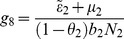
, 
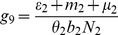
, 
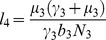
, 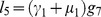.

Substituting equations (15) into equation (13) we obtain
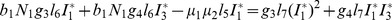
(18)where 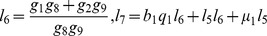.

In solving for the equilibria, we omit the expression containing 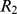 because it can be determined when 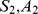 and 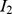 are known. We then determine analytically the conditions under which these equilibria are stable or unstable. The following result holds without proof to avoid repetition:

**Lemma 2.** *The resulting model is biologically relevant (solutions are positive) in the set*

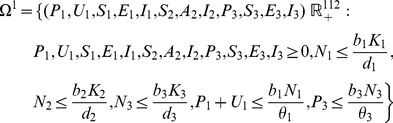
(19)


The model system (1,2,3) being nonlinear, stability analysis will be carried out via linearisation. The Jacobian matrix of system (1,2,3) at an arbitrary equilibrium is
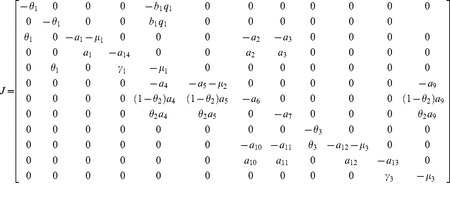
(20)where 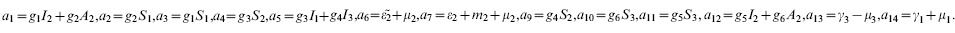.

Evaluating 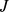 at the disease-free equilibrium and using basic properties of matrix algebra, it is evident from the characteristic polynomial of 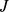 that the following eigenvalues 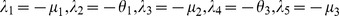 have negative real part and the remaining reduced matrix is
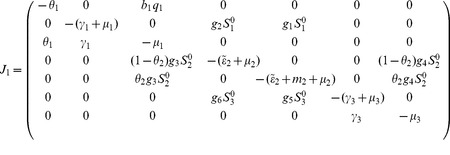
(21)


The stability of a disease-free equilibria should be established from the eigenvalues of the reduced Jacobian matrix (21). To simplify the computations, we perform the following operations on matrix (21): first we add the first row to the third one and take the resultant as the new third row; second we multiply the second row by 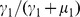 and add it to the new third row, then take the resultant as the new third row and at last we multiply the sixth row by 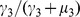 and add it to the last row and maintaining the rest as it is, we obtain the following matrix
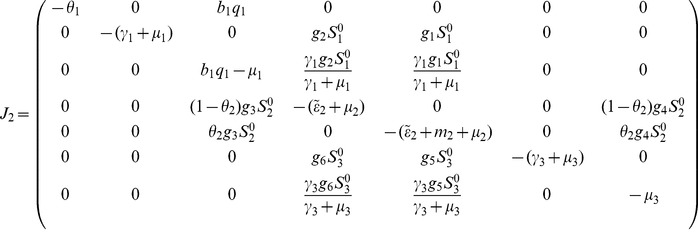
(22)


From the basic properties of matrix algebra, it is evident from the characteristic polynomial of 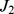 that the following eigenvalues 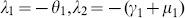 and 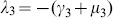 have negative real part and the remaining reduced matrix is
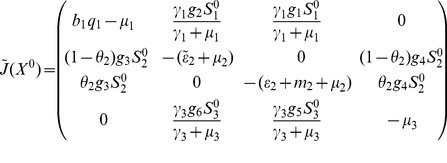
(23)


### 0.5 Stability analysis of the model (1,2,3) without *Culex* species

In the absence of *Culex* species, 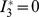, equation (18) can be written as
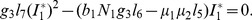
(24)


Equation (24) has two possible solutions 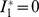 or 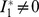. The case 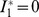 implies an existence of a disease-free equilibria and the case 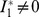 implies an existence of an endemic equilibria. Let us now derive conditions under which positive endemic equilibria exist. For 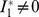, we get
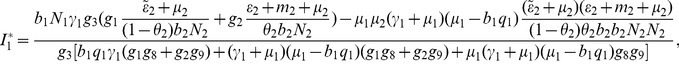
(25)



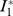
 is epidemiologically meaningful, that is, 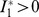 if and only if
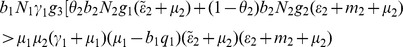
which can be written in the form
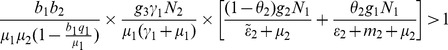
where 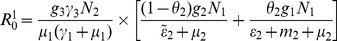 is the basic reproductive number for the model without *Culex* species and 
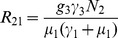
 represents the number of new infections in livestock from one infected *Aedes* mosquito and 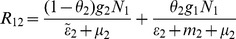 represent the number of new infections in *Aedes* mosquitoes from one infected (asymptomatic or symptomatic) animal and 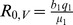 represents the vertical transmission reproductive number. Therefore, 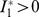 if and only if 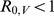 and 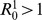. Thus, the following result holds:

**Theorem 1.** *The RVF model (1,2,3) without Culex species has exactly one disease-free equilibrium point (DFE),* 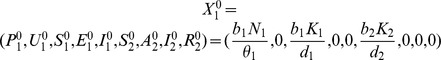 *for* 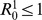 *and exactly one endemic equilibrium point (EE),* 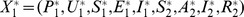 *whenever* 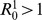 *.*

The result in Theorem 1 indicates the impossibility of backward bifurcation in the RVF model system (1,2,3) without *Culex* species since it has no endemic equilibrium when 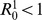. This explains that the model (1,2,3) without *Culex* species has a globally asymptotically stable disease-free equilibrium whenever 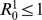.

In its simplest form, backward bifurcation in epidemic models usually implies the existence of two subcritical endemic equilibria when the basic reproductive number for 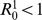, and a unique supercritical endemic equilibrium for 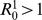 [37]. Thus, a unique positive endemic equilibrium exists only when 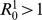. We note that the increase in complexity of an epidemic model (by adding more infected classes, for example) can lead to backward bifurcation and even more complicated phenomena associated with endemic equilibria [37]. However, increase in complexity of the proposed RVF model does not appear to give rise to more complex behaviour with regard to endemic equilibria.

#### 0.5.1 Local stability of DFE, 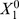


In the absence of secondary vector (*Culex* species) that serve as RVF outbreak amplifiers the Jacobian matrix 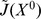 in (23) reduces to
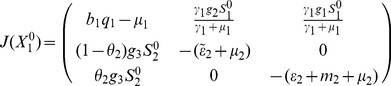
(26)


The characteristic equation corresponding to the above Jacobian matrix is
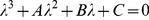
(27)where 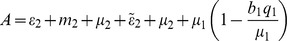, 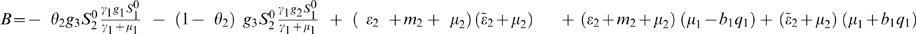, 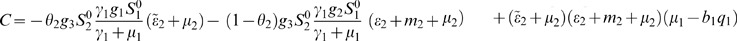


Here 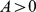 for 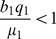, 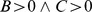 for 
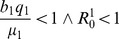
. Thus the equation (27) has no root which is positive or zero (Descartes' rule of sign). The equation (27) will only have negative roots or complex roots with negative real part if 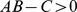 (according to Routh-Hurwitz criteria), that is, 
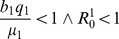
. Thus, the system (1,2,3) without *Culex* species is stable about the interior equilibrium 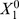 and the following result holds:

**Theorem 2.** *For* 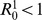 *the model system (1,2,3) without Culex mosquitoes has a unique DFE point which is locally asymptotically stable in* 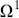 *.*

#### 0.5.2 Global asymptotic stability of DFE, 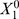


To ensure that the disease elimination is independent of the initial sizes of the populations, we need to show that the disease-free equilibrium 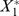 is globally asymptotically stable (GAS). This is established using the approach proposed in Castillo-Chavez et al. [38]. There are two conditions that if met guarantee the global asymptotic stability of the disease-free state. First, system (1,2,3) without *Culex* mosquitoes must be written in the form:
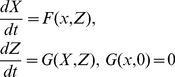
(28)where 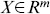 denotes (its components) the number of uninfected individuals and 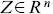 denotes (its components) the number of infected individuals including latent and infectious. 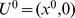 denotes the disease-free equilibrium of this system.

(H1) For 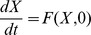, 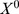 is globally asymptotic stable

(H2) 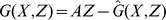, 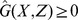 for 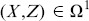
where 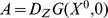 (see [31] for more details) is an M-matrix (the off diagonal elements of 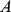 are nonnegative) and 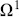 is the region where the model makes biological sense.

If the system (28) satisfies the above two conditions then the following Theorem holds.

**Theorem 3.** *The fixed point* 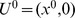 *is globally asymptotic stable equilibrium of system (28) provided that* 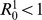 *(locally asymptotic stable) and that assumptions (H1) and (H2) are satisfied.*

**Proof 2.** *Rewriting the model system (1,2,3) without Culex mosquitoes in the form of* *equation (28* *) then* 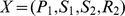 *,* 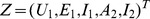 *and* 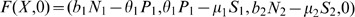 *, then*

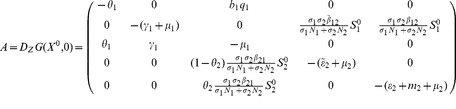
(29)and 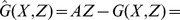




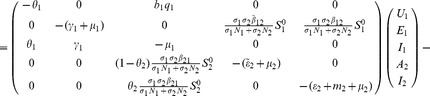


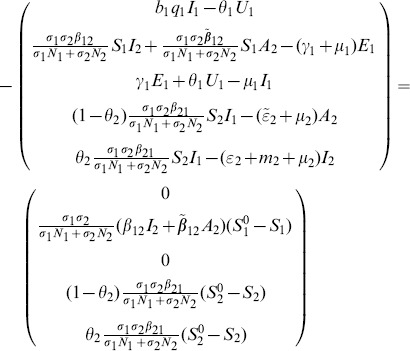



*Since* 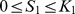 *and* 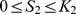 *it is clear that* 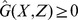 *. Then* 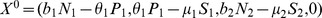 *is globally asymptotic stable equilibrium of* 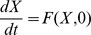 *. Hence, by the above Theorem,* 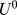 *which represents the disease-free equilibrium* 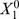 *is globally asymptotic stable.*

#### 0.5.3 Global asymptotic stability of EE, 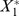


Since the DFE is locally stable when 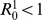 (this will suggest local stability of the EE for the reverse condition [32]), we only investigate the global stability of the endemic equilibrium.

**Theorem 4.** *For* 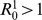 *, the model system (1,2,3) without Culex mosquitoes has unique positive EE point* 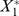 *, such that*


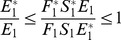
 *for* 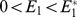,


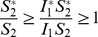
 *for* 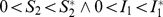 *and*


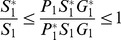
 *for* 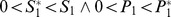


*Then,* 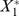 *is globally asymptotic stable in* 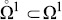 *.*

**Proof 3.** *Global stability of the EE is explored via the construction of a suitable Lyapunov function. Let us consider the following function:*

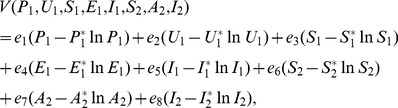
(30)
*where* 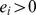 *for* 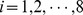 *with* 
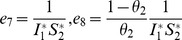
 *.* 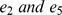 *are chosen very small such* 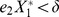 *,* 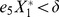 *for* 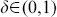 *. V (* 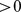 *in* 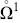 *) is a Lyapunov function (Korobeinikov* [*39*] *). The time derivative of V is*



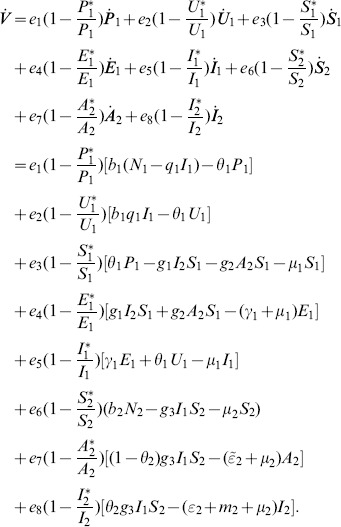
(31)


*At* 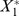 *, we have* 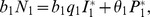 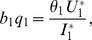 
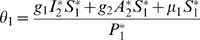
, 
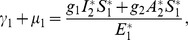
 
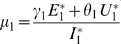
, 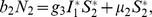 
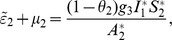
 
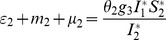
.

*Let* 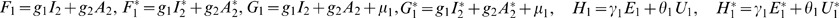. *Then,* 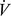 *can now be written as*

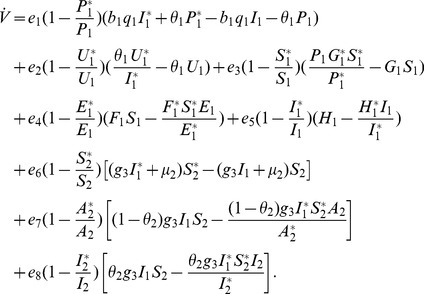
(32)


*Further simplification yields*

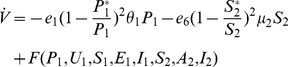
(33)
*where*

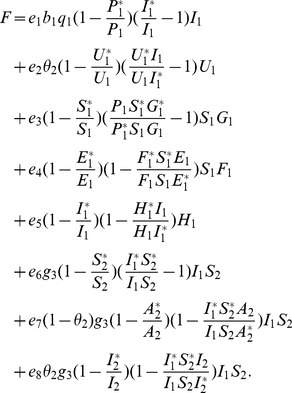
(34)


*Recalling that* 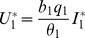 *,* 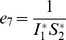 *and* 
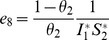
 *we obtain,*

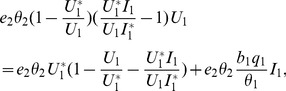
(35)

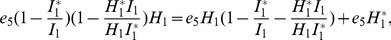
(36)
*and* 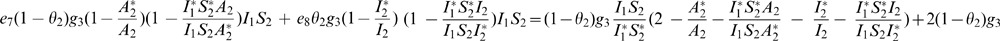.

*By theorems hypothesis,*

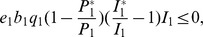


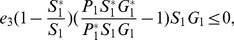


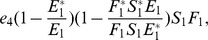


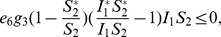

*where strict equalities holds only when,*

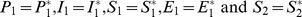
.

*Furthermore,*

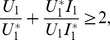


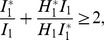


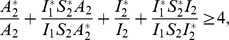

*for all* 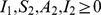 *, because the arithmetic mean is greater than or equal to the geometric mean. Thus,* 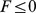 *for* 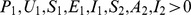 *. Hence,* 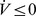 *for all* 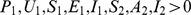 *and is equal to zero for* 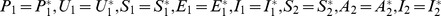 *and* 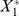 *is the only equilibrium state of the system on this plane. Therefore, the largest compact invariant set in* 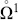 *such that* 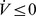 *is the singleton* 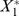 *which is the endemic equilibrium point. LaSalle's invariant principle* [*40*] *guarantees that* 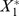 *is globally asymptotically stable (GAS) in* 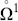 *, the interior of* 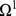 *.*

### 0.6 Stability analysis of the overall model (1,2,3)

The overall model system (1,2,3) describes the epidemiological and ecological complexity involved on RVF dynamics. Theorem 2 in van den Driesche and Watmough [32] states that the local stability of the disease-free equilibrium of the model can be determined by its basic reproduction number, 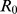. However, in host-vector models where multiple transmission cycle are observed to occur as in the case of our model (vertical transmission, host to *Aedes* infection, *Aedes* to host infection, host to *Culex* infection and *Culex* to host infection) the basic reproductive number obtained via next-generation method does not give the number of host infected by a single host if there an intermediate vector, but rather the geometric mean of the number of infections per generation [41]. Therefore, in our case the local stability of the disease -free equilibrium, 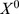, (10) of the model is established through the Routh-Hurtwitz criteria [42], [43], and the following result holds.

**Theorem 5.** *The model system (1,2,3) always has the disease-free equilibrium* 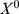 *. If* 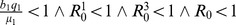 *, the disease-free equilibrium is locally asymptotically stable in* 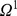 *.*

**Proof 4.** *To prove the stability of the equilibrium point* 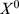 *we use the Jacobian matrix (23) of the linearised system, which yield the following characteristic polynomial:*

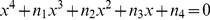
(37)
*where* 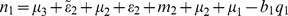, 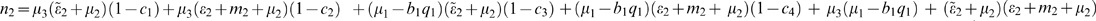, 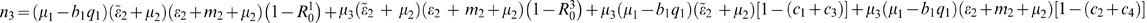, 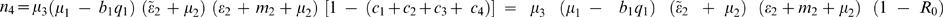, *with* 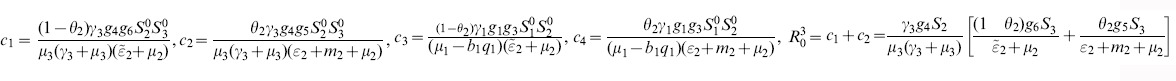 *.*

*Thus,* 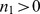 *for* 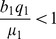 *,* 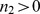 *for* 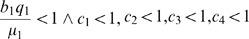 *,* 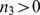 *for* 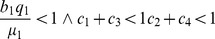 *and* 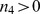 *for* 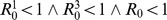 *. Thus the* *equation (37* *) has no root which is positive or zero (Descartes' rule of sign). Therefore* *equation (37* *) will only have negative roots or complex roots with negative real part if* 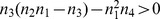 *(according to Routh-Hurwitz criteria), that is,* 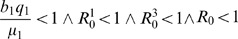 *. Thus, the system (1,2,3) is locally asymptotically stable about the interior equilibrium* 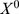 *.*

#### 0.6.1 Existence and uniqueness of endemic equilibrium, 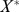


The existence of the endemic equilibrium in 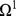, is determined by equation (18). Taking 
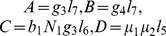
 and 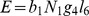, equation (18) can be written as
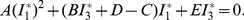
(38)


Solving equation (38) for 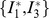 we get 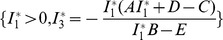 which gives 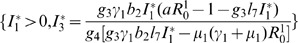, with 
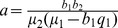
. The existence of positive 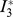 is given by the following inequalities: 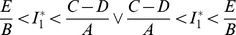.

Since 
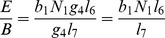
 and 
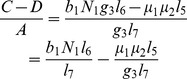
, we get that the meaningful inequality is 
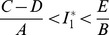
, thus 
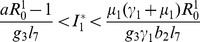
.

Since 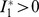, then 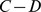 should be positive. 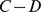 is the expression on the numerator of equation (25), which was verified to be positive whenever 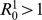 and 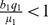. This gives the threshold for the endemic persistence. Therefore the following result holds:

**Theorem 6.** *The RVF model (1,2,3) has a unique endemic equilibrium point* 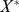 *whenever* 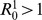 *and* 
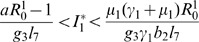
 *.*

**Figure 2:**
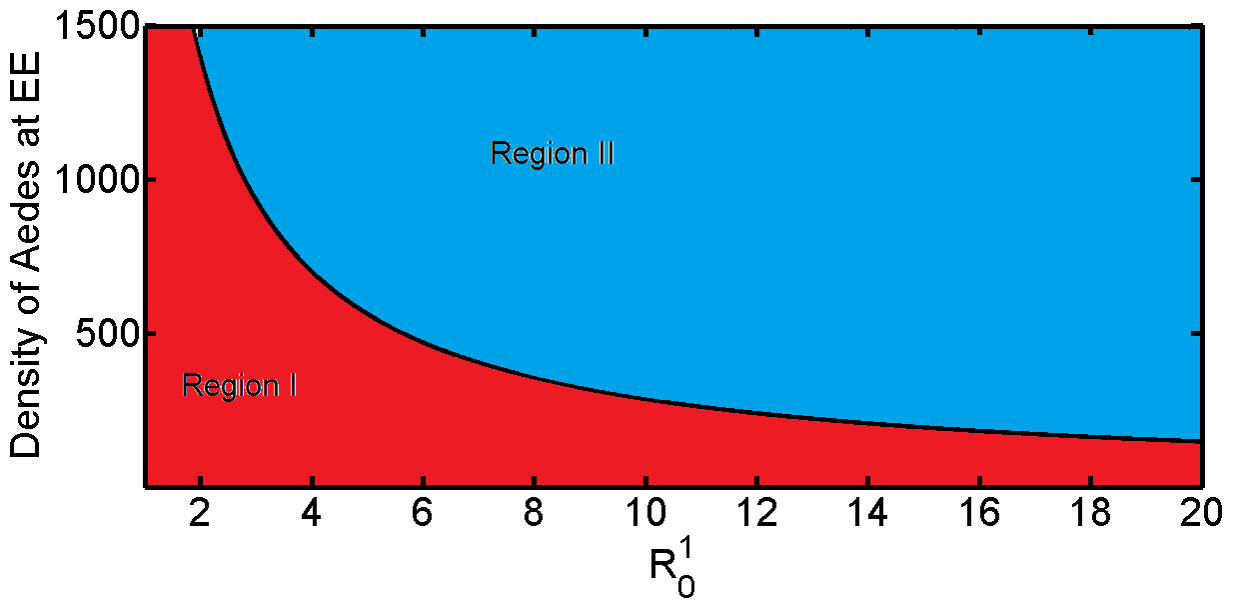
Based on equation (38), we represent the condition for existence of infected *Culex* mosquitoes at the endemic equilibrium (EE) state. The existence of infected *Culex* is impossible in region I. In region II both *Aedes* and *Culex* coexist. The border black line represents the threshold of coexistence, which is exactly 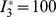.

The result in Theorem (6) indicates that depending on vertical transmission efficiency, if the *Aedes* basic reproduction number 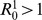 and 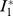 satisfy the inequality 
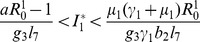
, it is sufficient to cause an outbreak, since secondary vectors (*Culex* species) co-exist and serve as disease amplifiers. Figure 2 shows the region where 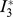 is strictly positive when varying both 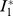 and 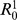. That is, in region II both infected *Aedes* and *Culex* co-exist while in region I only infected *Aedes* exist. This confirm the analytical results obtained above. The existence of infected *Culex* at endemic equilibrium depend on the existence infected *Aedes* and initial spread of the disease 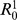. Thus, *Aedes* species have the potential to initiate the epidemic through transovarial transmission and the potential to sustain low levels of the disease during post epidemic periods.

### 0.7 Bifurcation and chaos investigation on the RVF model

**Figure 3:**
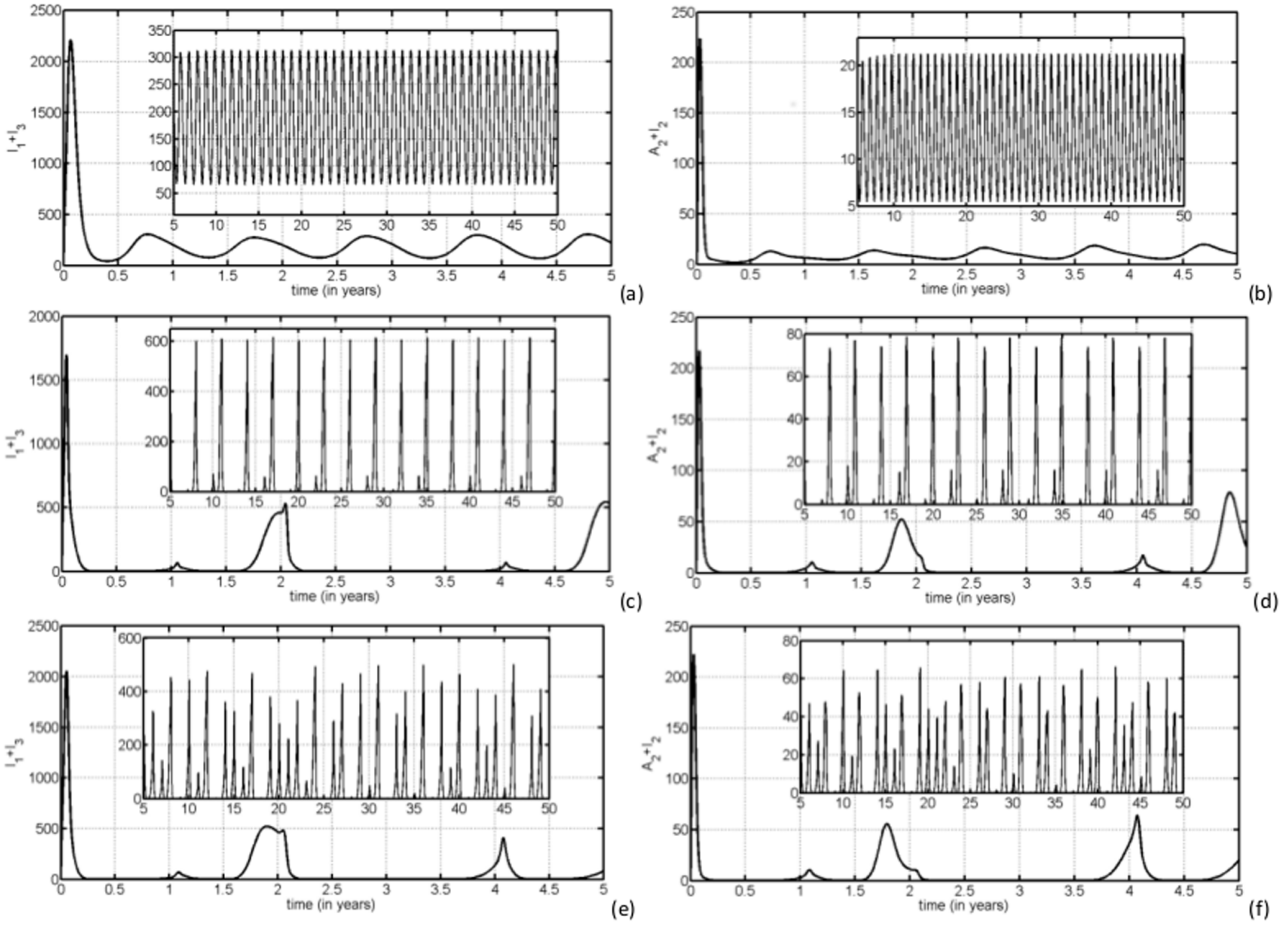
We display the time series of (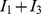) left and (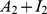) right. Parameters used for (a) and (b) are 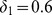, 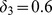, for (c) and (d) are 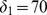, 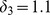, finally for (e) and (f) are 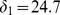, 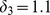. Figures (d) and (f) show a linear increase in livestock seroprevalence during post-epidemic which comes in cycles of 5 to 7 years approximately.

**Figure 4:**
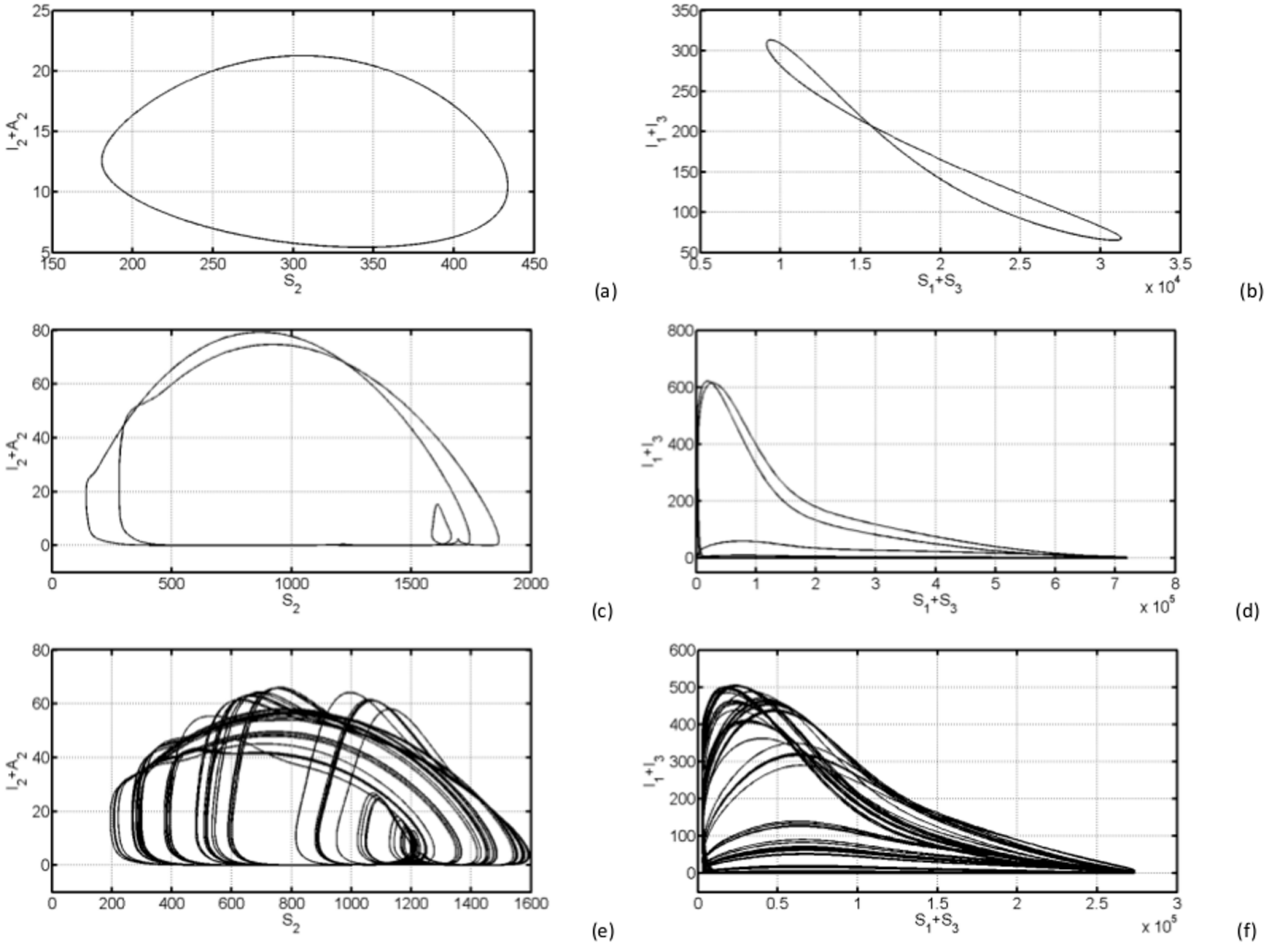
Phase portrait with couple (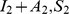) on the left and (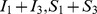) on the right. In (a) and (b), 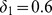, 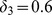, the system is attracted by a limit cycle. In (c) and (d), 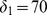, 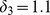, the system is multi-periodic. And in (e) and (f), 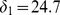, 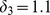, the systems behave with higher multi periodicity.

To provide some numerical evidence for the qualitative dynamic behaviour of the model (1,2,3), time series with both transient and permanent regimes, phase portraits, Poincaré maps, bifurcation diagrams, Lyapunov exponents have been used to assess model sensitive dependence on initial conditions and return maps are used to illustrate the above analytical results and for determining new dynamics as the parameters vary. We start by introducing a simple case of seasonality on time dependent oviposition rates of mosquito populations (*Aedes* and *Culex*):
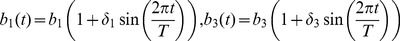
(39)


where 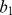 and 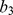 are the baseline parameters of the oviposition rates of *Aedes* and *Culex* mosquitoes respectively, 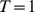 year, 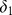 and 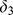 are the external forcing amplitudes for the two species of mosquitoes respectively, which represent the strength of seasonality that controls the magnitude of the fluctuations. When 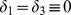, the model reduces to a non-seasonal model and the system possesses two types of equilibria: disease free and endemic equilibria. When the magnitude of the external forcing parameters 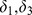 is sufficiently small, 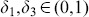 the system responds with oscillations of the same annual period as external forces (see Figs.3 (a) and (b)). However with larger values (for instance 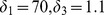) the system shows other modes of oscillations (see Figs.3 (c) and (d)) with period 5 as confirmed by Poicaré maps Fig.4. In all this section, the system is integrated numerically with the fifth order Runge-Kutta algorithm [44]. The initials conditions and other values are 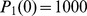, 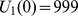, 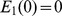, 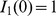, 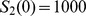, 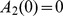, 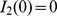, 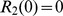, 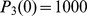, 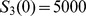, 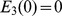, 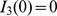, 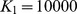, 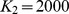 and 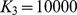. The parameter values are shown in Table 2.

#### 0.7.1 Time series simulations

Figure 3 depicts the time evolution of the sum of infectious *Aedes* and *Culex* mosquitoes, 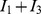 and sum of infectious asymptomatic and symptomatic livestock for different values of 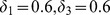; 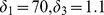 and 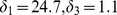. In (a) the number of infectious mosquitoes oscillates yearly reaching the same maximum. In (c) the quantity 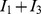 also oscillates with first peak of above 500 around the second year. In (c) we notice a long lasting peak of about 500 infectious mosquitoes in the interval 18–25 months, which is likely to cause an inter-epidemic outbreak. Fig.3(b) shows a constant low oscillation, high peaks around second and fifth year in (d) and high peaks around second and fourth year in (f). Note that the internal figures describes the permanent regime which represent the dynamics where the system is expected to adapt to the external forcing. The time series for 
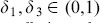
, also show that the total of infected vectors 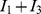 and infected livestock 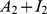 stay quite away from zero, avoiding the chance of extinction in stochastic system with reasonable size (see Figs.3 (a) and (b)). This is due to the fact that for 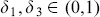 vector oviposition continues throughout the year, albeit at lower rates during unfavourable seasons. This is not the case of East African region, where we have two rainy seasons (long and short) and a dry season, where under this former we expect stochastic extinction during some intervals of inter-epidemic periods.

In the region 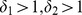, Figs.3 (c)–(f), we observe fluctuations in the total number of infected from reasonable small peaks (describing RVF post-epidemic activities) to very low values, which in this case drive almost surely the system to extinction.

#### 0.7.2 Phase portrait diagrams and Poincaré maps

Instead of studying the entire complicated trajectories, important information is encoded in the phase plane. This approach allow us to analyse geometrically the total dynamics of the system. Varying 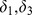 the state space plots show a rich dynamical behaviour with bifurcations from limit cycles, multi-periodic oscillation to completely irregular behaviour which is usually the fingerprint of chaos (see Fig.4).

Poincaré map is a useful tool for analysing the dynamics of a nonlinear system. It allows good insight for global dynamics of the system by displaying the types of attractors of the system [45]. The successive iterations of the map are defined as:
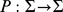


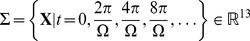
(40)


**Figure 5:**
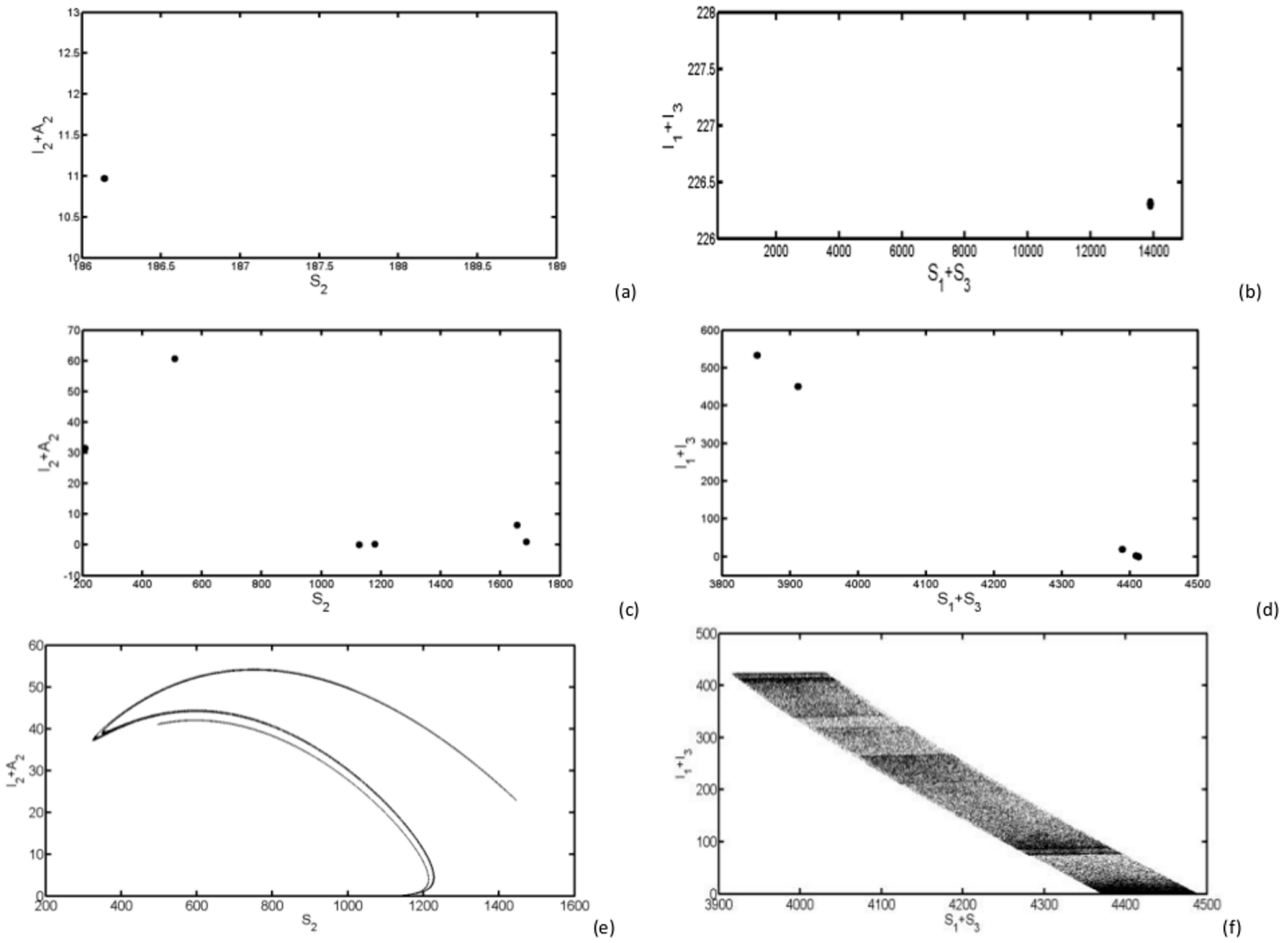
Poincaré maps with couple (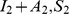) on the left and (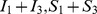) on the right. In (a) and (b), 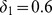, 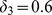, in (c) and (d), 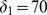, 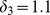 and in (e) and (f), 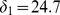, 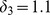.

The attractor is generated by sampling the system stroboscopically at time corresponding to the multiple of the period 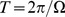. We have used 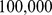 points and a period of one year. Figures 5 (a) and (b) with (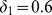, 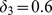) show that the system is attracted by a limit cycle, because of the presence of a single dot. In this case the system is periodic. In (c) and (d) with (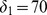, 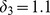) we notice a presence of a few dots, thus, the system is multi-periodic and in (e) and (f) with (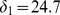, 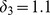) we notice a strange attractor which is usually a sign of a chaotic system.

#### 0.7.3 Maxima return maps of 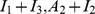 for state phase plots

**Figure 6:**
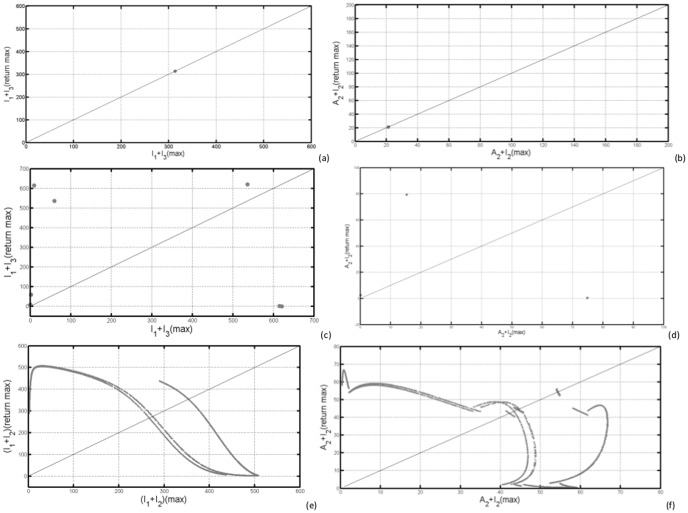
We display the maxima return map of 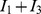 and 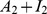 with (a)–(b) 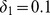, 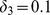, (c)–(d) 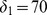, 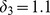 and (e)–(f) 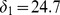, 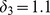. The blanc line represents the first bisectrix of the plane.

We have used maxima return maps in order to get supplementary classification of different dynamics for parameters 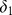 and 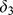. For a time selected as 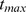, at which 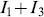 and 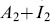 have a local maximum, we have plotted the number of infected mosquitoes and livestock respectively at time 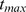 and at the next local maximum 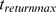. Figures 6 (a) and (b) show that all consecutive maxima coincide with themselves as shown by a single dot. In (c) and (d), we notice that consecutive maxima are few and different as a sign of irregularity, and in (e) and (f), we observe that a dot rarely comes back to the same point. Thus, the fingerprint of chaotic attractor is clearly visible now with the maxima return maps analysis.

#### 0.7.4 Lyapunov exponents and bifurcation diagrams

The largest Lyapunov Exponent (LE) is quantitatively characterized by the average rate of separation of infinitesimally close trajectories in the phase space for a dynamic system. It can be used to determine how sensitive a dynamical system is to initial conditions [46]. In general for a N-dimensional dynamical system described by a set of equations 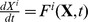, the LEs are defined by [35]:
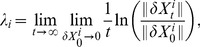
(41)


where 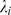 is the 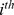 LE and 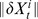 is the distance between the trajectories of the 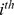 component of the vector field 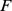 at time 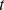. Recall that exponential divergence in the phase space is given by the LEs. If the largest LE is less than or equal to zero, then the system may be regarded as periodic or quasi-periodic. Otherwise, if the largest LE is positive the system may have an irregular or chaotic behaviour. Another important fact to be mentioned is that negative LE does not, in general, indicate stability, and that positive largest LE does not, in general indicate chaos [47].

**Figure 7:**
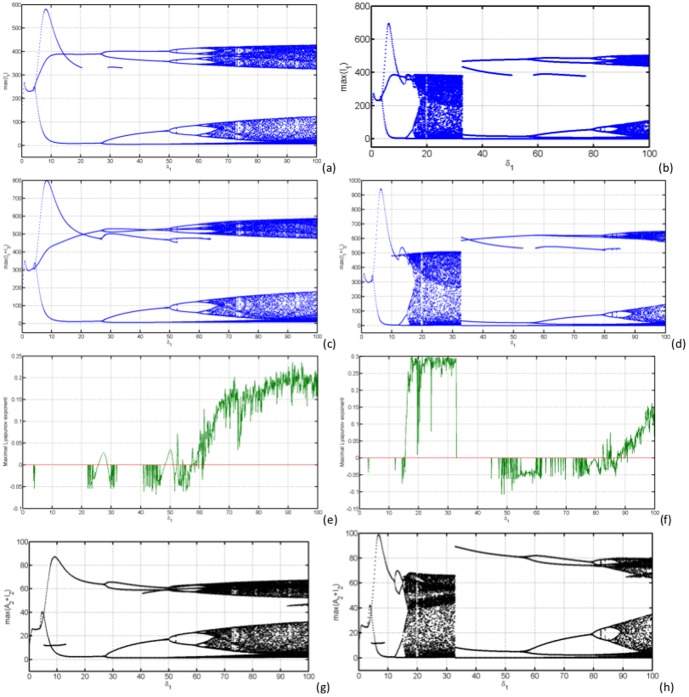
In (a) and (b), bifurcation diagrams for the local maximal quantities of 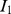 by varying the parameter 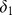 and fixing 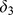 = 0.1(a) and 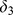 = 1.1(b). In (c) and (d), bifurcation diagrams for the local maximal quantities of 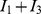 by varying the parameter 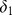 and fixing 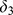 = 0.1(c) and 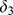 = 1.1(d). In (e) and (f), we have computed the largest LE for 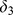 = 0.1(e) and 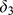 = 1.1(f) and in (g) and (h), bifurcation diagrams for the local maximal quantities of 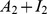 by varying the parameter 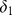, and fixing 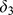 = 0.1(h) and 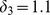
(f).

In Figs.7 (a)–(d) we have computed the bifurcation diagrams with respect to 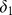, the external forcing amplitude on the response of the RVF model. Figures (e) and (f) show the maximal LE after infinitesimal perturbation of 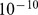 in the initial conditions. In Fig.7(e), the maximal LE is positive for 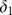 ≳ 60 and around 50 and 25. In Fig.7(f), the maximal LE is positive for 15 ≲ 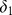 ≲ 34 and for 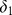 ≳ 85.

Figure 7 shows the bifurcation diagrams of the local maxima of infectious mosquitoes and livestock undergoing forward forking bifurcation from period-1 to period-6 oscillatory type behaviour. In Fig.7(a), local maxima extrema 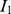 of infectious *Aedes* species undergo irregular behaviour for 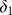 ≳ 65, which is the fingerprint of chaos. Fig.7(b) shows irregular behaviour for 15 ≲ 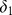 ≲ 34 and 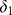 ≳ 85, with large number of periods. In Figs.7 (c) and (d), we observe almost the same qualitative behaviour with the same parameters, but with notable difference in the value of the local maxima of the overall infectious mosquitoes fuelled by the elevation of several secondary vectors which serve as disease amplifiers. When 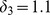 the local extrema 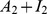 undergoes irregular behaviour for 15 ≲ 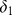 ≲ 34 and 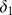 ≳ 85, with large number of periods Fig.7(h).

We observe from Fig.7(e) that for a fixed 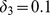 and varying 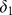 (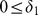 ≲ 62) the largest Lyapunov exponent is fairly negative indicating stable limit cycles and multi-periodicity with some shift to positive values as the system bifurcates through period doubling routes to chaos. Above 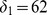 a positive Lyapunov exponent clearly moves away from zero, indicating deterministically chaotic attractors. For a fixed 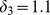 and varying 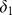 Fig.7(f) the largest Lyaponov exponent fairly confirms the behaviour seen through bifurcation diagrams with positive values on the chaotic regions.

#### 0.7.5 Interaction between *Culex* and *Aedes* oviposition rates

**Figure 8:**
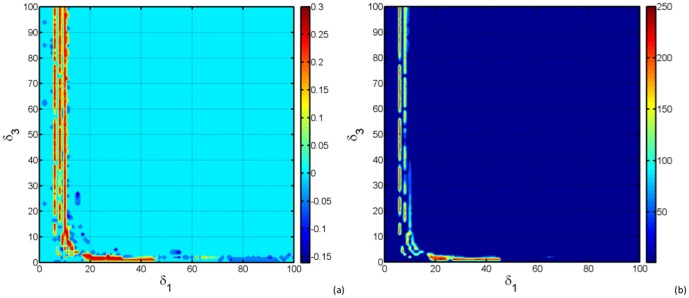
In (a) we display the maximal LE function of 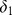 and 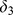. The colorbar shows the value of the maximal LE. In (b) we display the number of points in the Poincaré map (the colorbar) according to the set of parameters (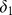, 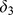).

In the preceding section we have fixed the value of 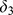, while investigating the bifurcation behaviour when 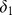 is varying. In Figure 8 we have computed the maximal LE when those two parameters are varying. For 20 ≲ 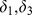 ≲ 100, the maximal LE is negative, then the system is sensitive to initial conditions. For low values of 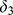 and 18 ≲ 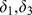 ≲ 45, the maximal LE is positive. Another remarkable fact is observed when 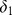 is around 10 no matter the value of 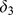, the maximal LE will be positive. This shows i fact that, *Aedes* oviposition rate is predominant in leading irregular behaviour in our system, confirming that *Aedes* are indeed the RVF primary vectors.

Both maximal Lyapunov exponent functions of 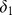 and 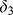 and the Poincaré map of the set (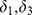) fig.8 around 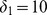 agree with each other, confirming the analytical results obtained in Theorem 6.

Recall that in certain *Aedes* species of the subgenera *Neomelaniconion* and *Aedimorphus*, the female mosquitoes transmit RVF virus vertically to their eggs [3]. When these mosquitoes lay their eggs in flooded areas, transovarially infected adults may emerge and transmit RVF virus to nearby domestic animals which may then lead to the infection of secondary arthropod vectors species including various *Culex* [48]. Thus, there is an initial quantity of primary infected vectors required to trigger an outbreak. Fig.8 shows that if the control magnitude of fluctuations in *Aedes* oviposition rate is around 10, and the number of newly transovarially infected mosquitoes is amplified by nearby domestic animals, then, the number of infected (in both host and vector) will be sufficiently enough to cause subsequent elevation of secondary vectors, including *Culex* species, and consequently trigger an outbreak.

## Discussion and Conclusion

The proposed model accounts for the population dynamics of both livestock and mosquitoes (*Aedes* and *Culex*) and seasonal changes in weather that heavily affects the vector population size. Mosquito density varies over seasons, and the contact rates and vector oviposition rates vary dynamically based upon both host and vector densities since female mosquitoes need blood for oviposition. Qualitative analysis of the model showed that there exists a domain where the model is epidemiologically and mathematically well-posed. We then analysed the existence and stability of both disease free and endemic equilibria.

Dynamical analysis shows that when 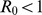, then the disease dies out and when 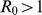 the disease become endemic. A suitably constructed Lypunov function is used to determine the global stability of the endemic equilibrium of the model without *Culex* species and the existence of the endemic equilibrium of the overall model is seen to exist whenever 
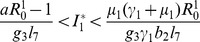
, meaning that the co-existence of the infectious host, *Aedes* and *Culex* mosquitoes is subjected to the number of infected *Aedes* mosquitoes.

We have used visualisation techniques to study the behaviour of RVF epidemic model under external forcing in the mosquito oviposition rates. The bifurcation diagrams show the emergence with increase in external forcing parameters 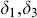 of Hopf and pitchfork modes of bifurcation. That they have much larger amplification of infection levels that can take place if the system is encouraged to switch to multi-periodic mode. In transition, further amplification can occur if the multi-periodic mode becomes unstable and the system moves into chaotic state before finding an alternative stable periodic mode (e.g. Fig.7).

On the bifurcation diagrams the highest maximum number of infectious *Aedes* mosquitoes is only observed for values of 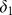 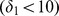 with different values of 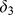, meaning that for the disease to trigger an inter-epidemic a certain number of infectious *Aedes* mosquitoes is necessary. This confirm the analytical results obtain in section 0.6, as well as results obtained in [9] which showed that when mosquito populations follow seasonal patterns with large amplitudes, vertical transmission could play a significant role in long-term persistence of a pathogen. Another important conclusion is that even with a low maximum number of infectious individuals, the bifurcation diagrams show that if for fixed 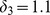 and varying 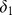 the system becomes chaotic in the interval 15 ≲ 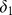 ≲ 35, meaning that unpredictable and possibly uncontrolled low levels of inter-epidemic activities may occur, leading to higher morbidity in livestock. Hence observed fluctuations in RVF outbreak data and non deterministic nature of RVF inter-epidemic activities could now be better understood considering fluctuations on both rainy and dry season as significant factor.

A sero-survey study done in livestock approximately four years after the 2006/07 RVF outbreak in Tanzania, showed a linear increase in seroprevalence in the post-epidemic annual cohorts implying a constant exposure and presence of active foci transmission [10]. Figure 3 (d) and (f) demonstrate this behaviour which is shown to come in cycles of 5 to 7 years approximately, as well as fluctuations in the total number of infected from reasonable small peaks (describing RVF post-epidemic activities) to very low values. During these periods of low troughs for the total number of infected, the virus survive through vertical transmission in *Aedes* species and among wild animals as reservoirs [49]. Note that, this recurrent low level RVF virus activity during inter-epidemic periods, in East African region in particular, infects 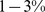 of livestock herds annually [50]. Generally, these infections pass undetected where there is no regular active surveillance in the livestock and human populations [10]. This suggests that RVF outbreaks partly result from build up RVF inter-epidemic activities for it has been observed that optimum climatic conditions (temperature and rainfall) only and presence of mosquitoes can not completely explain the RVF outbreaks [51].

Simulation of the interaction between the two populations densities of *Aedes* and *Culex* by varying the magnitudes of external forcing 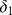 and 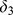 of the oviposition rates 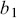 and 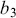 have opened a new window of research on the potential of *Aedes* species to initiate RVF outbreaks and sustain low endemic levels of the disease during inter-epidemic periods. This result concurs with the Chitnis et al. [9] suggestion that vertical transmission is required for inter-epidemic persistence.

One of the main objectives of this study was to investigate the possibility of prediction of RVF outbreaks with the aim of controlling RVF incidence. We have shown that seasonality may induce irregular behaviour on the disease dynamics. It has been shown that the interaction between oviposition rates of *Aedes* and *Culex* mosquitoes makes prediction more complex. In fact, higher irregularity are naturally expected in the higher seasonality forcing. However, our proposed model has shown that the complexity occurs even for a relatively low level of the magnitude of seasonal forces. We have also found that seasonal *Aedes* oviposition rate is most likely to generate uncontrollable behaviour than *Culex* seasonal oviposition rate. This study is of great epidemiological significance as it highlights a high uncertainty in RVF outbreak prediction by a simple theoretical mathematical model including seasonal influence in mosquito populations. In addition, the model including external seasonal forcing on mosquito oviposition rates shows ability to mimic the linear increase in livestock seroprevalence as reported in Sumaye et al. [10], with first post-epidemic peak around the second year, a following peak larger than the previous one around the fifth year (see Fig.3 (d) and (f)).

Currently, two types of RVF vaccine for animals exist: a live vaccine and inactivated vaccine. However, the current live vaccine can not be used for prevention and prevention using the inactivated vaccine is almost impossible to sustain in RVF affected countries for economic reasons [6], [21], [52]. Then, the possible alternative of controlling RVF transmission remains in keeping the vector population at the lowest levels. Therefore we argue that locations that may serve as RVF virus reservoirs should be eliminated or kept under control to prevent multi-periodic outbreaks and consequent chains of infections. We also recommend a systematic surveillance in the livestock or human population in order to monitor inter-epidemic RVF activities.

This study is not exhaustive and can be extend to include humans not just as dead ends [18] but also as disease amplifiers since it has been demonstrated that humans have potential to transmit the virus, particularly to *Aedes* mosquito species [8]. Also, including ticks on the model may help to explain and gain more insights on the understanding of disease dynamics and enhance control strategies, since ticks have been reported to play a role on disease transmission [51]. For mathematical convenience and tractability of the model, we made several assumptions, thus our results are driven by the model formulation and structure. A step toward a more quantitative and qualitative study is viable by relaxing some of the assumptions made and incorporating more epidemiological features of the disease as well as the use of a double periodic function and inclusion of stochasticity in order to capture the dynamic of the two rainfall seasons in East Africa (long and short rainy seasons), where the disease is likely to be more predominant. Further studies are needed to enhance the understanding of RVF epidemic and inter-epidemic activities in order to provide further insights in assessing the current and future control strategies.

## Supporting Information

Appendix S1 A1. Computation of the basic reproduction number. A2. Computation of the basic reproduction number in periodic environments.
(PDF)
