# Supplementary material for: Stability, Bifurcation and Chaos Analysis of Vector-Borne Disease Model with Application to Rift Valley Fever
Source: PLoS One. 2014 Oct 1;9(10):e108172. doi: 10.1371/journal.pone.0108172 (PMC4182743; doi:10.1371/journal.pone.0108172)
Supplement: Appendix S1 — A1. Computation of the basic reproduction number. A2. Computation of the basic reproduction number in periodic environments. (PDF) [file pone.0108172.s001.pdf]

Stability, bifurcation and chaos analysis of vector-borne disease model with application to Rift Valley fever

Sansao A. Pedro, Shirley Abelman, Frank T. Ndjomatchoua, Rosemary Sang, Henri E.Z. Tonnang
Methods S1: Supporting Information

APPENDIX A

A-1 Computation of the basic reproduction number

First, we calculate the basic reproduction number for the vertical transmission route, $R_{0,V}$. For this case, the only compartments involved are the infected eggs, exposed adults, and infectious adults of the *Aedes* population. Thus we have, in the notation of reference [1],

$$\frac{d}{dt}\begin{pmatrix} U_1 \\ E_1 \\ I_1 \end{pmatrix} = F_v - V_v = \begin{pmatrix} 0 \\ 0 \\ \theta_1 U_1 \end{pmatrix} - \begin{pmatrix} \theta_1 U_1 - b_1 q_1 I_1 \\ \gamma_1 E_1 + d_1 \frac{E_1 N_1}{K_1} \\ d_1 \frac{I_1 N_1}{K_1} - \gamma_1 E_1 \end{pmatrix} \tag{A-1}$$

The corresponding Jacobian matrices at the disease free equilibrium of the above system are

$$F_v^0 = \begin{pmatrix} 0 & 0 & 0 \\ 0 & 0 & 0 \\ \theta_1 & 0 & 0 \end{pmatrix}, \quad V_v^0 = \begin{pmatrix} \theta_1 & 0 & -b_1 q_1 \\ 0 & \gamma_1 + \mu_1 & 0 \\ 0 & -\gamma_1 & \mu_1 \end{pmatrix} \tag{A-2}$$

The basic reproduction number for the vertical transmission is calculated as the spectral radius of the next generation matrix, $(F_v V_v^{-1})$

$$R_{0,V} = \frac{b_1 q_1}{\mu_1} = \frac{b_1 q_1 K_1}{d_1 N_1}.$$

Next, we calculate the horizontal transmission basic reproduction number, $R_{0,H}$. For this mode of transmission we must evaluate the exposed and infectious compartments of the *Aedes*, *Culex* and asymptomatic and infectious compartments of the livestock populations. To simplify the calculation of R_0, we transform our system to consider the percent of the population made up by each compartment, $x_i = \frac{X_i}{N_i}$, where X_i is a compartment of population i,

$$\frac{d}{dt}\begin{pmatrix} e_1 \\ i_1 \\ a_2 \\ i_2 \\ e_3 \\ i_3 \end{pmatrix} = F_H - V_H =$$

$$\begin{pmatrix} l_1\beta_{12} i_2 N_2(1-e_1-i_1) + l_1\tilde{\beta}_{12} a_2 N_2(1-e_1-i_1) \\ 0 \\ (1-\theta_2) l_1 \beta_{21} i_1 N_1 (1-a_2-i_2-r_2) + (1-\theta_2) l_3 \beta_{23} i_3 N_3 (1-a_2-i_2-r_2) \\ \theta_2 l_1 \beta_{21} i_1 N_1 (1-a_2-i_2-r_2) + \theta_2 l_3 \beta_{23} i_3 N_3 (1-a_2-i_2-r_2) \\ l_3 \beta_{32} i_2 N_2 (1-e_3-i_3) + l_3 \tilde{\beta}_{32} a_2 N_2 (1-e_3-i_3) \\ 0 \end{pmatrix} - \begin{pmatrix} (\gamma_1+d_1)e_1 \\ b_1 i_1 - \gamma_1 e_1 - \theta_1 u_1 \\ (\tilde{\varepsilon}_2 + d_2) a_2 - m_2 i_2 a_2 \\ (\varepsilon_2 + d_2 + m_2) i_2 - m_2 (i_2)^2 \\ (\gamma_3 + d_3) e_3 \\ b_3 i_3 - \gamma_3 e_3 \end{pmatrix} \tag{A-3}$$

where $l_1 = \dfrac{\sigma_1\sigma_2}{\sigma_1 N_1 + \sigma_2 N_2}$ and $l_3 = \dfrac{\sigma_3\sigma_2}{\sigma_3 N_3 + \sigma_2 N_2}$.

The corresponding Jacobian matrices at the disease free equilibrium of the above system are:

$$F_H^0 = \begin{pmatrix} 0 & 0 & l_1^0\tilde{\beta}_{12}N_2^0 & l_1^0\beta_{12}N_2^0 & 0 & 0 \\ 0 & 0 & 0 & 0 & 0 & 0 \\ 0 & (1-\theta_2)l_1^0\beta_{21}N_1^0 & 0 & 0 & 0 & (1-\theta_2)l_3^0\beta_{23}N_3^0 \\ 0 & \theta_2 l_1^0\beta_{21}N_1^0 & 0 & 0 & 0 & \theta_2 l_3^0\beta_{23}N_3^0 \\ 0 & 0 & l_3^0\tilde{\beta}_{32}N_2^0 & l_3^0\beta_{32}N_2^0 & 0 & 0 \\ 0 & 0 & 0 & 0 & 0 & 0 \end{pmatrix},$$

$$V_H^0 = \begin{pmatrix} \gamma_1 + b_1 & 0 & 0 & 0 & 0 & 0 \\ \gamma_1 & b_1 & 0 & 0 & 0 & 0 \\ 0 & 0 & \tilde{\varepsilon}_2 + b_2 & 0 & 0 & 0 \\ 0 & 0 & 0 & \varepsilon_2 + b_2 + m_2 & 0 & 0 \\ 0 & 0 & 0 & 0 & \gamma_3 + b_3 & 0 \\ 0 & 0 & 0 & 0 & -\gamma_3 & b_3 \end{pmatrix} \tag{A-4}$$

where $l_1^0 = \dfrac{\sigma_1\sigma_2}{\sigma_1 N_1^0 + \sigma_2 N_2^0}$ and $l_3^0 = \dfrac{\sigma_3\sigma_2}{\sigma_3 N_3^0 + \sigma_2 N_2^0}$.

The spectral radius of $F_H^0(V_H^0)^{-1}$ is given by:

$$R_{0,H} = \sqrt{\frac{(1-\theta_2)(l_3^0)^2\beta_{23}\tilde{\beta}_{32}\gamma_3 N_2^0 N_3^0}{b_3(\tilde{\varepsilon}_2+b_2)(\gamma_3+b_3)} + \frac{(1-\theta_2)(l_1^0)^2\beta_{21}\tilde{\beta}_{12}\gamma_1 N_1^0 N_2^0}{b_1(\tilde{\varepsilon}_2+b_2)(\gamma_1+b_1)} + \frac{\theta_2(l_3^0)^2\beta_{23}\beta_{32}\gamma_3 N_2^0 N_3^0}{b_3(\varepsilon_2+b_2+m_2)(\gamma_3+b_3)} + \frac{\theta_2(l_1^0)^2\beta_{21}\beta_{12}\gamma_1 N_1^0 N_2^0}{b_1(\varepsilon_2+b_2+m_2)(\gamma_1+b_1)}} \tag{A-5}$$

When strictly defined as the reproductive rate of the pathogen, R_0 for the overall model that accounts for both vertical infection and horizontal transmission is given by:

$$R_0 = \frac{b_1 q_1}{2\mu_1} + \frac{1}{2}\sqrt{R_{0,V}^2 + 4R_{0,H}^2} \tag{A-6}$$

A-2 Computation of the basic reproduction number in periodic environments

The concept behind the definition of R_0 in a periodic environment as the spectral operator on space of periodic functions is described below, and for more details see [2,3].

For all $t \in R$ and $x \geq 0$, let $K(t,x)$ be a nonnegative $n \times n$ matrix. Assume that $K(t,x)$ is a periodic function of t of period α for all $x \geq 0$. The idea behind the function $K(t,x)$ is an epidemic model with n "infected" compartments $(I_1, I_2, \cdots, I_n)$, which may be infectious or latent. The coefficient $K_{i,j}(t,x)$ in row i and column j represents the expected number of individuals in compartment I_i that one individual in compartment I_j "generates" at the beginning of an epidemic per unit time at time t if it has been in compartment I_j for x units of time. The verb 'generates' cover the case where individuals in compartment I_j infect individuals in compartment I_i, but also the case where individuals in compartment I_j just move to compartment I_i [2].

By linearizing the system $(1,2,3)$ near the disease-free equilibrium point

$$(\frac{b_1 N_1}{\theta_1}, 0, \frac{b_1 K_1}{d_1}, 0, 0, \frac{b_2 K_2}{d_2}, 0, 0, 0, \frac{b_3 N_3}{\theta_3}, \frac{b_3 K_3}{d_3}, 0, 0)$$

we have:

$$
\begin{aligned}
\dot{U}_1(t) &= b_{s1}(t)q_1 I_1 - \theta_1 U_1 \\
\dot{E}_1(t) &= \frac{\sigma_1\sigma_2\beta_{12}}{\sigma_1 N_1 + \sigma_2 N_2} I_2 S_1^0 + \frac{\sigma_1\sigma_2\tilde{\beta}_{12}}{\sigma_1 N_1 + \sigma_2 N_2} A_2 S_1^0 - (\gamma_1 - d_1\frac{N_1}{K_1})E_1 \\
\dot{I}_1(t) &= \gamma_1 E_1 + \theta_1 U_1 - d_1\frac{I_1 N_1}{K_1}, \\
\dot{A}_2(t) &= (1-\theta_2)\frac{\sigma_1\sigma_2\beta_{21}(t)}{\sigma_1 N_1 + \sigma_2 N_2} I_1 S_2^0 + (1-\theta_2)\frac{\sigma_3\sigma_2\beta_{23}(t)}{\sigma_3 N_3 + \sigma_2 N_2} I_3 S_2^0 - (\tilde{\varepsilon}_2 - d_2\frac{N_2}{K_2})A_2 \\
\dot{I}_2(t) &= \theta_2\frac{\sigma_1\sigma_2\beta_{21}(t)}{\sigma_1 N_1 + \sigma_2 N_2} I_1 S_2^0 + \theta_2\frac{\sigma_3\sigma_2\beta_{23}(t)}{\sigma_3 N_3 + \sigma_2 N_2} I_3 S_2^0 - (\varepsilon_2 + m_2 + d_2\frac{N_2}{K_2})I_2 \\
\dot{E}_3(t) &= \frac{\sigma_3\sigma_2\beta_{32}}{\sigma_3 N_3 + \sigma_2 N_2} I_2 S_3^0 + \frac{\sigma_3\sigma_2\tilde{\beta}_{32}}{\sigma_3 N_2 + \sigma_2 N_2} A_2 S_3^0 - (\gamma_3 + d_3\frac{N_3}{K_3})E_3 \\
\dot{I}_3(t) &= \gamma_3 E_3 - d_3\frac{I_3 N_3}{K_3},
\end{aligned}
\tag{A-7}
$$

The transmissibility number $\bar{R}_0$ is defined through the spectral radius of a linear integral operator on a space of periodic functions, thus the operator $K(t,x)$ is given by:

$$
\begin{pmatrix}
0 & 0 & b_1 q_1 e^{-\mu_1 x} & 0 & 0 & 0 & 0 \\
0 & 0 & 0 & l_1^0\tilde{\beta}_{12}S_1^0 e^{-v_1 x} & l_1^0\beta_{12}S_1^0 e^{-v_2 x} & 0 & 0 \\
\theta_1 e^{-\theta_1 x} & \gamma_1 e^{-v_3 x} & 0 & 0 & 0 & 0 & 0 \\
0 & 0 & (1-\theta_2)l_1^0\beta_{21}S_2^0 e^{-\mu_1 x} & 0 & 0 & 0 & (1-\theta_2)l_3^0\beta_{23}S_2^0 e^{-\mu_3 x} \\
0 & 0 & \theta_2 l_1^0\beta_{21}S_2^0 e^{-\mu_1 x} & 0 & 0 & 0 & \theta_2 l_3^0\beta_{23}S_2^0 e^{-\mu_3 x} \\
0 & 0 & 0 & l_3^0\tilde{\beta}_{32}S_3^0 e^{-v_1 x} & l_3^0\beta_{32}S_3^0 e^{-v_2 x} & 0 & 0 \\
0 & 0 & 0 & 0 & 0 & \gamma_3 e^{-v_4 x} & 0
\end{pmatrix}
\tag{A-8}
$$

where $v_1 = (\tilde{\varepsilon}_2 + \mu_2),\ v_2 = (\tilde{\varepsilon}_2 + m_2 + \mu_2), v_3 = (\gamma_1 + \mu_1),\ v_4 = (\gamma_3 + \mu_3)$.
Thus the integral operator G_j gives:

$$
\begin{aligned}
G_j = {} & \frac{b_1 q_1}{\mu_1 + 2\pi ji} \bullet \frac{\theta_1}{\theta_1 + 2\pi ji} + \frac{\gamma_1}{\gamma_1 + \mu_1 + 2\pi ji} \bullet \frac{(l_1^0)^2\beta_{21}S_2^0 S_1^0}{\mu_1 + 2\pi ji}\left[\frac{(1-\theta_2)\tilde{\beta}_{12}}{\tilde{\varepsilon}_2 + \mu_2 + 2\pi ji} + \frac{\theta_2\beta_{12}}{\varepsilon_2 + m_2 + \mu_2 + 2\pi ji}\right] \\
& + \frac{\gamma_3}{\gamma_3 + \mu_3 + 2\pi ji} \bullet \frac{(l_3^0)^2\beta_{23}S_3^0 S_2^0}{\mu_3 + 2\pi ji}\left[\frac{(1-\theta_2)\tilde{\beta}_{32}}{\tilde{\varepsilon}_2 + \mu_2 + 2\pi ji} + \frac{\theta_2\beta_{32}}{\varepsilon_2 + m_2 + \mu_2 + 2\pi ji}\right]
\end{aligned}
\tag{A-9}
$$

References

1. van den Driessche P and Watmough J (2002) Reproduction numbers and sub-threshold endemic equilibria for compartmental models of disease transmission. Math. Biosci. 180(1): pp. 29-48.

2. Bacaer N (2007) Approximation of the basic reproduction number R0 for vector-Borne viseases with a periodic vector population. Bull. Math. Biol. 69: 1067-1091.

3. Grassly NC and Fraser C (2006) Seasonal infectious disease epidemiology. Proc. R. Soc.273: 2541-2550.
